# tRNA engineering strategies for genetic code expansion

**DOI:** 10.3389/fgene.2024.1373250

**Published:** 2024-03-07

**Authors:** YouJin Kim, Suho Cho, Joo-Chan Kim, Hee-Sung Park

**Affiliations:** Department of Chemistry, Korea Advanced Institute of Science and Technology, Daejeon, Republic of Korea

**Keywords:** tRNA engineering, genetic code expansion, directed evolution, rational design, unnatural amino acid

## Abstract

The advancement of genetic code expansion (GCE) technology is attributed to the establishment of specific aminoacyl-tRNA synthetase/tRNA pairs. While earlier improvements mainly focused on aminoacyl-tRNA synthetases, recent studies have highlighted the importance of optimizing tRNA sequences to enhance both unnatural amino acid incorporation efficiency and orthogonality. Given the crucial role of tRNAs in the translation process and their substantial impact on overall GCE efficiency, ongoing efforts are dedicated to the development of tRNA engineering techniques. This review explores diverse tRNA engineering approaches and provides illustrative examples in the context of GCE, offering insights into the user-friendly implementation of GCE technology.

## 1 Introduction

Genetic code expansion (GCE) is an innovative technique that allows for the introduction of unnatural amino acids (UAAs) into proteins, surpassing the constraints of the natural genetic code. This technique extends beyond the conventional set of 20 amino acids, creating new opportunities to engineer proteins with unique chemical, structural, and functional properties. The core of GCE lies in its ability to redefine or expand the genetic alphabet, by identifying specific positions in messenger RNA’s open reading frame through nonsense codons, quadruplet codons, and rare sense codons. The technique was inspired by naturally occurring nonsense suppressor transfer RNAs (tRNAs) in the 1990s. Early efforts involved *in vitro* aminoacylation of orthogonal suppressor tRNAs with desired UAAs (Hecht and Chinault, 1976; Hecht et al., 1978; Heckler et al., 1984), subsequently incorporated into proteins through both *in vitro* (Bain et al., 1989; Bain et al., 1991a; Bain et al., 1991b; Noren et al., 1989) and *in vivo* translation (Liu et al., 1997a). However, the limitations of *in vitro* aminoacylation-based systems spurred the quest for more efficient methods, leading to the development of GCE systems utilizing orthogonal aminoacyl-tRNA synthetase/tRNA pairs in host cells since the early 2000s (Wang et al., 2001).

**FIGURE 1:**
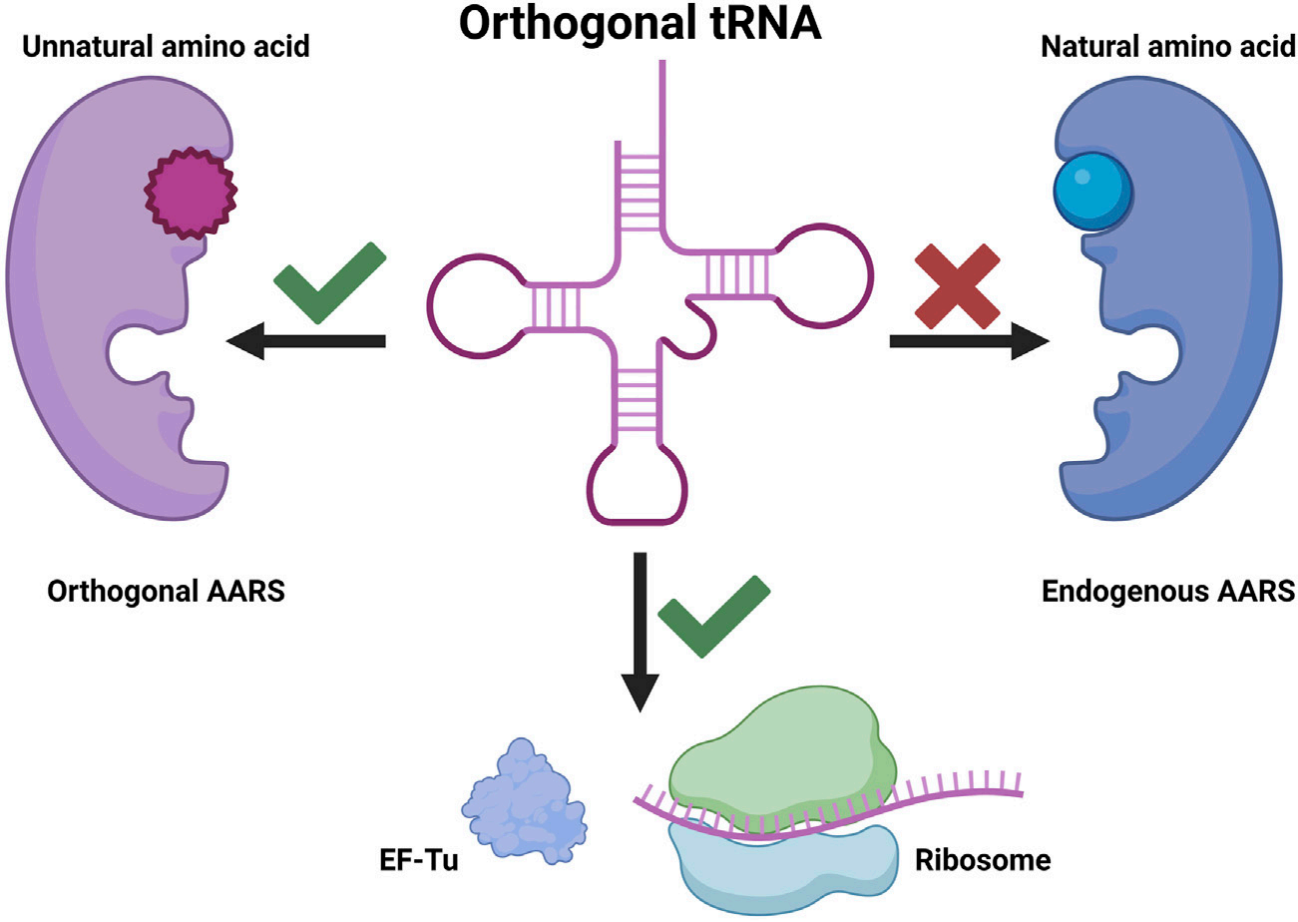
Orthogonal tRNA as a fundamental element in genetic code expansion.

Genetic code expansion (GCE) is based on three key components: aminoacyl-tRNA synthetase (AARS), tRNA, and UAA. In GCE experiments, a specific AARS charges UAA onto a particular tRNA (usually a nonsense suppressor tRNA), and the aminoacylated tRNA is introduced into the endogenous translation system. It is essential for the AARS to charge the desired UAA exclusively onto the specific tRNA during this process. Moreover, the tRNA should not be charged by endogenous AARSs to prevent mis-incorporation. An AARS and tRNA pair that exhibits these characteristics is commonly referred to as an orthogonal AARS/tRNA pair (Figure 1). The advancement of GCE has been achieved through various efforts to identify orthogonal AARS/tRNA pairs capable of incorporating diverse UAAs. Typically, organisms of different phyla are used to obtain orthogonal AARS/tRNA pairs, and additional directed evolution is often performed to enhance orthogonality and incorporation yield (Melnikov and Söll, 2019).

While early efforts mainly focused on AARS evolution for UAA charging, the expansion of host cells from *Escherichia coli* to other species highlighted the challenges in maintaining the efficiency of AARS/tRNA pairs across different hosts. To provide stability and utility within the host cell environment, tRNAs for GCE must satisfy two seemingly conflicting criteria: orthogonality to host cell AARSs and cooperativity with the rest of cellular machinery. Consequently, tRNA evolution emerged as a formidable challenge in the development of GCE techniques, aiming not only to increase orthogonality within host cells but also to enhance overall incorporation efficiency by working seamlessly with the host cell’s transcriptional and translational machinery. This review explores various tRNA engineering techniques for GCE, delving into the intricacies of tRNA’s structure, function, and binding partners. We will also discuss notable cases where tRNA engineering has played a significant role in advancing this transformative technology.

## 2 The structure of tRNA and its binding partners

tRNA is as an essential molecule within the intricate cellular machinery, serving as a bridge between the genetic code encoded in DNA and the synthesis of proteins-the fundamental building blocks of life. Its multifaceted interactions with key cellular partners, including ribosome, AARS, and elongation factor (EF), position tRNA as a central role in translating genetic information into functional proteins. A comprehensive understanding of tRNA’s structure, its association with binding partners, and the specific recognition mechanisms governing these interactions is essential for unraveling the intricacies of protein synthesis and its regulatory processes.

### 2.1 The structure of tRNA

**FIGURE 2:**
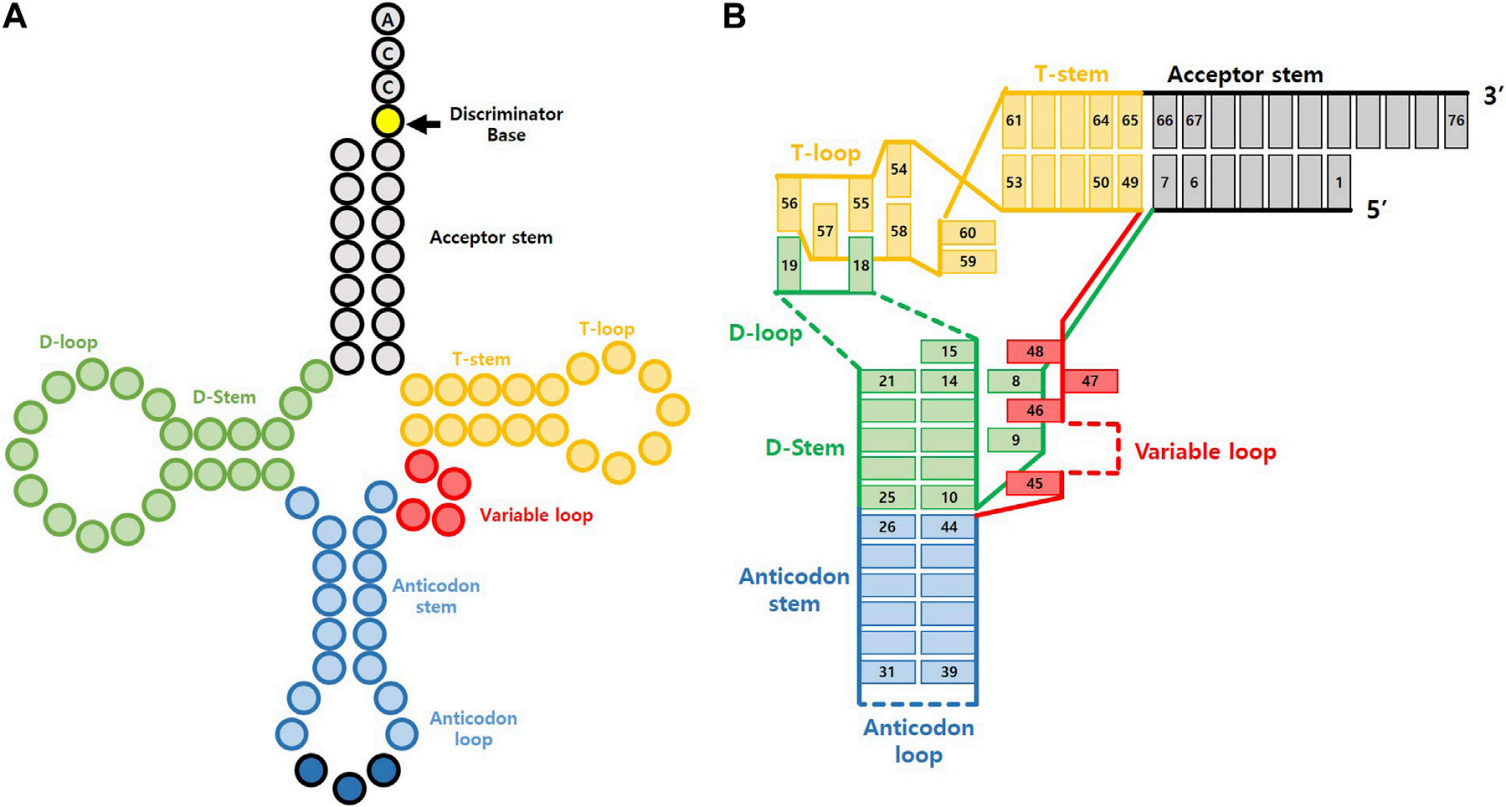
General structure of tRNA. **(A)** Cloverleaf representation **(B)** L-shape tRNA representation with base pairing.

tRNA is a short RNA molecule that contains 76 to 90 ribonucleotides. It folds into a specific structure, as illustrated in Figure 2A, which includes the acceptor stem, the D arm, the anticodon arm, the variable arm, the T arm, and the 5′ terminal CCA sequence (Sharp et al., 1985). Its structure was first discovered in 1965 when researchers isolated yeast alanyl tRNA, sequenced it, and used base pairing information to propose a 2-D cloverleaf model (Holley et al., 1965). Later, this model’s reliability was confirmed using NMR experiments (Bolton and Kearns, 1975), and X-ray crystallography revealed the actual 3-D structure to be L-shaped, as depicted in Figure 2B (Kim et al., 1974). This L-shaped structure is highly conserved in nearly all organisms (Giegé et al., 2012), but the sequences and sizes of each domain can vary. In some cases, specific domains like D arm or T arm are entirely omitted, as observed in certain mitochondrial tRNA or tRNA from particular species (de Bruijn et al., 1980).

The L-shaped structure of tRNA can be categorized into two branches based on the bending point, known as the elbow. The acceptor branch includes the acceptor stem and a part of the T arm. This is the area where a specific amino acid is charged onto tRNA by AARS. The length and composition of the acceptor branch vary according to the organism. On the other hand, the anticodon branch is located opposite the acceptor branch, and it includes the D arm and the anticodon arm. This branch helps match mRNA on the ribosome, which allows the synthesis of specific amino acids into proteins. Meanwhile, the variable loop varies greatly in length and composition among species. It plays a crucial role in distinguishing numerous tRNAs by recognizing specific binding partners. For a more in-depth exploration of tRNA structure, readers are recommended to refer to a recent review (Berg and Brandl, 2021).

### 2.2 Interactions with binding partners of tRNA

**FIGURE 3:**
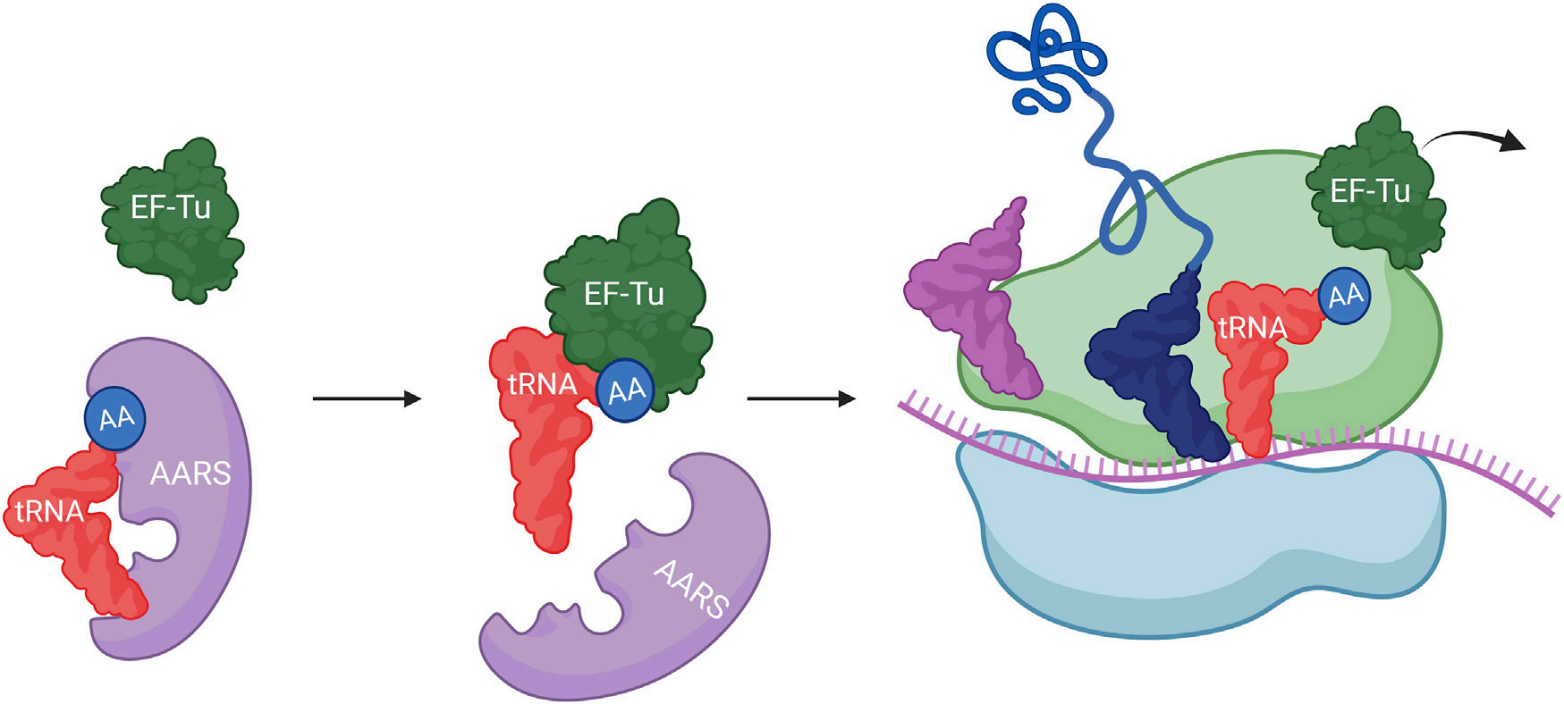
The role of tRNA in translation.

The involvement of tRNA in protein expression initiates with the charging of amino acids at the 2′ or 3′ hydroxyl end by AARS. Then, the aminoacylated tRNA, which has an anticodon complementary to the codon, is transported to the reaction center in the ribosome by EF-Tu (in prokaryotes) or EF-1α (in eukaryotes), leading to sequential peptide synthesis. During the translation process, tRNA interacts with various binding partners, including AARS, EF-Tu, and ribosomes (Figure 3). Each binding partner interacts with specific positions on tRNA through hydrogen bonding with nucleobases. Thus, modifying the known binding sites on tRNA could be advantageous in altering interactions between tRNA and specific components during the protein expression process.

**FIGURE 4:**
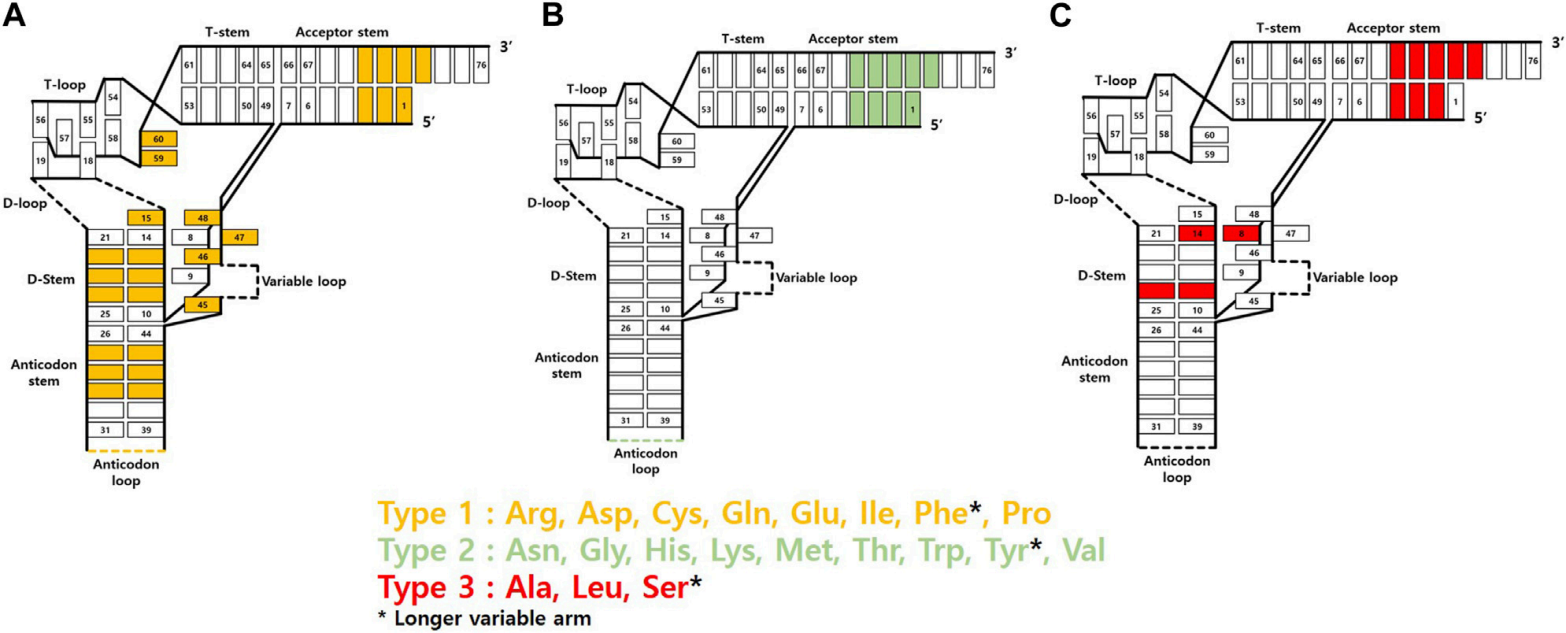
AARS interaction sites of tRNA based on AARS types. **(A)** Type 1 AARSs **(B)** Type 2 AARSs **(C)** Type 3 AARSs.

The most notable binding partner of tRNA in the GCE is the corresponding AARS. The way it interacts with tRNA varies depending on its type and origin. Essentially, as the primary function of AARS is to attach amino acids to the 3′ end of tRNA, all AARS types exhibit interactions with the 3′ end of tRNA. Additionally, the most frequent interaction sites within tRNA, often situated at the 5′ end of the D stem or in the vicinity of the 3′ end of the acceptor stem, have been identified (Tamaki et al., 2018). Various AARS binding sites show unique structural elements for each tRNA, allowing them to be distinguished by AARS. These specific sites act as identity elements, enabling precise acylation of different tRNAs present in organisms. Among these sites, the anticodon loop and the 73rd discriminator base contain the most abundant identity elements. Except for SerRS, AlaRS, LeuRS, and PylRS, all AARS possess anticodon recognition ability. Similarly, the 73rd base in the acceptor stem, being close to the 3′ end where the actual ligation takes place, was thought to be a favorable site for AARS recognition (Saks et al., 1994; Ho et al., 2018). However, it is now known that only tRNAs acylated with ten specific amino acids possess identity elements through acceptor stem interactions with AARS. Furthermore, in the case of seryl, phenylalanyl, and tyrosyl tRNAs, tRNAs have variable loops with different lengths and shapes, allowing each AARS to be recognized uniquely (Figure 4). Hence, tRNAs exhibit a range of AARS interaction sites, which can offer significant potential for the generation of diverse orthogonal AARS/tRNA pairs.

**FIGURE 5:**
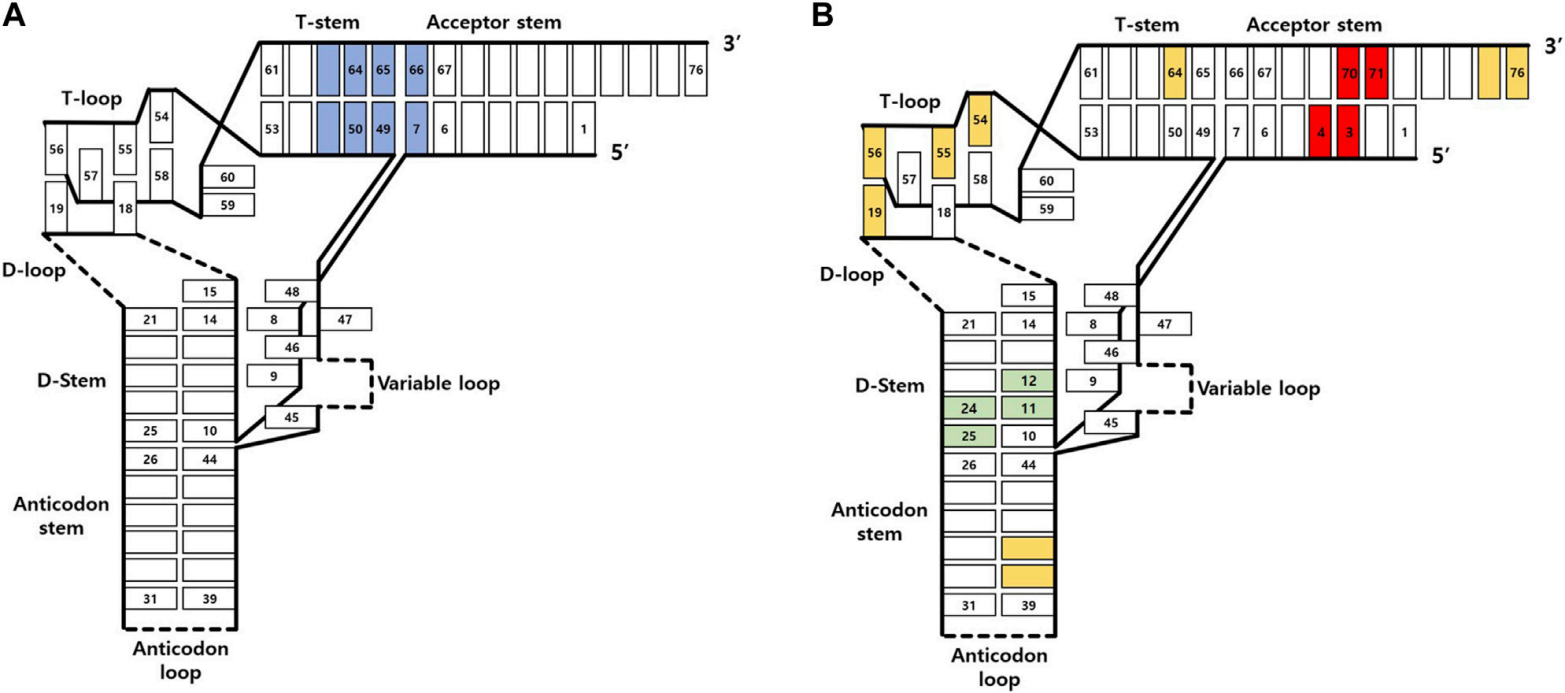
Depiction of tRNA interaction sites with **(A)** EF-Tu and **(B)** the ribosome. The regions interacting with EF-Tu are marked in blue, while those interacting with the A site, P site, and E site of the ribosome are illustrated in yellow, green, and red, respectively.

The interaction between EF-Tu and tRNA, along with the critical tRNA bases involved in these interactions, has been elucidated through NMR-based structural analysis (Förster et al., 1993), X-ray crystallography (Nissen et al., 1999; Eargle et al., 2008), and tRNA mutagenesis (Schrader et al., 2009). Specifically, the pairs 51:63, 50:64, 49:65, and 7:66 in the acceptor stem and T stem play a crucial role in this interaction (Figure 5A). However, it is noteworthy that these findings are limited to the bacterial system, and whether the same tRNA sites interact with EF in eukaryotic systems or archaea remains to be investigated.

The ribosome features three tRNA binding sites known as the A, P, and E sites, and tRNA engages with the ribosome in a distinctively specific manner at each of these binding sites (Yusupov et al., 2001; Voorhees et al., 2009; Demeshkina et al., 2010) (Figure 5B). With the aid of EF-Tu, aminoacylated tRNA makes its way into the A site through anticodon-codon recognition. The anticodon loop region, elbow region, and 3′ end of tRNA interact with the ribosome to secure the binding of approved aminoacylated tRNA. Once acylated tRNA moves into the P site, tRNA stabilizes its interaction in a position similar to that in the A site. Moreover, the D stem region plays a part in guiding tRNA-ribosome binding and supporting the peptidyl transfer reaction through ribosomal interaction at the P site. After peptidyl transfer, at the E site, the acceptor stem region facilitates the dissociation of tRNA from the ribosome by interacting with ribosomal RNA.

## 3 tRNA engineering strategies

### 3.1 Directed evolution of tRNA

**FIGURE 6:**
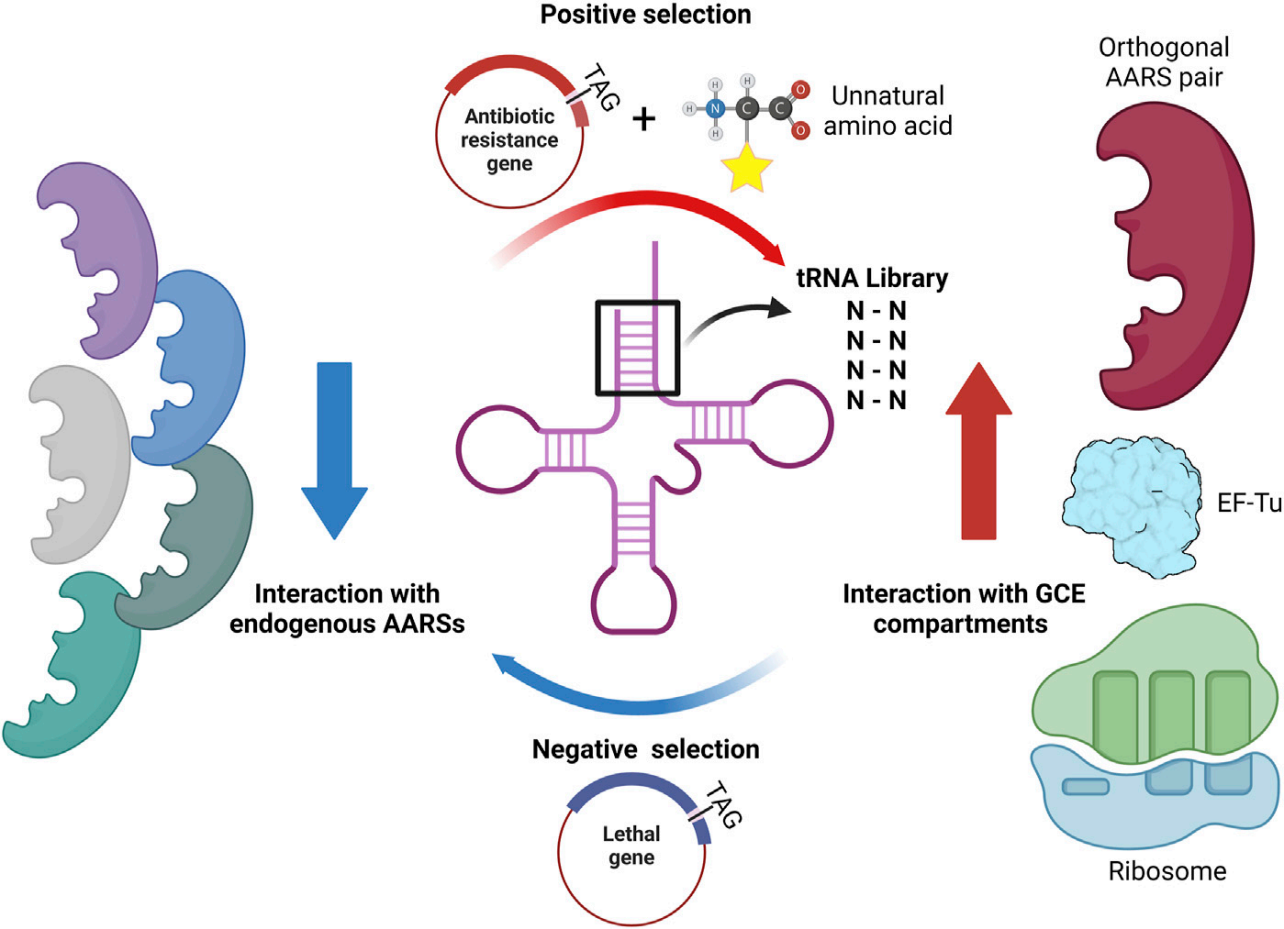
Directed evolution of tRNA through library generation and screening.

In 2001, the Schultz group developed a novel approach to create orthogonal AARS/tRNA pairs by utilizing the *Methanocaldococcus jannaschii* (Mj) TyrRS/tRNA pair for efficient UAA incorporation (Wang et al., 2001; Wang and Schultz, 2001). To enhance tRNA orthogonality, they implemented *in vivo* selection, which involved positive selection with β-lactamase and negative selection with barnase (Figure 6). In the process of positive selection, the desired UAA is added to the growth medium, allowing for the identification of active tRNAs that can prevent premature termination of translation when the stop codon is introduced in the antibiotic resistance gene. To eliminate tRNAs that are aminoacylated by endogenous AARS, negative selection is employed with the use of a lethal gene containing stop codons in the absence of UAA. This approach helped researchers to identify a tRNA molecule that could be charged by Mj TyrRS but not by *E. coli* (Ec) AARSs. This orthogonal tRNA was chosen from a random library comprising 11 sites of the Mj tRNA^Tyr^_CUA_ that do not interact with Mj TyrRS. This innovative strategy paved the way for the widespread adoption of screening orthogonal AARS/tRNA pairs through directed evolution. The primary objectives of tRNA evolution are to enhance orthogonality with AARSs of host cells, to improve cooperativity with the translational machinery, and to increase the stability of the tRNA molecule. The strategy for tRNA evolution varies based on the specific objective of each study, involving the creation of libraries targeting particular regions of tRNA and subsequent screening (Figure 6). This section will explore studies focusing on a particular region(s) of tRNA targeted for directed evolution.

#### 3.1.1 Acceptor stem engineering

The acceptor stem of tRNA interacts with the CP domain or Rossmann-fold domain of AARS (Choi et al., 2003). In this region, a directed evolution was conducted to diminish the interaction between the tRNA and the host cell’s AARSs while enhancing its interaction with the orthogonal AARS pair, thereby improving orthogonality and translation efficiency. The increase in orthogonality was obtained by screening an acceptor stem library of *Tetrahymena thermophila* tRNA^Gln^_CUA_ (Rodriguez et al., 2007). Researchers were able to generate amber (CUA) and opal (UCA) suppressor tRNAs that are orthogonal to eukaryotic cells. Incorporation of various tryptophan analogs into green fluorescent protein (GFP) in *E. coli* using the *Saccharomyces cerevisiae* (Sc) TrpRS/tRNA^Trp^ pair was achieved by acceptor stem library screening (Chatterjee et al., 2013a). To obtain orthogonal tRNA variants with enhanced activity, researchers first introduced a U68C mutation into the Sc tRNA^Trp^_CUA_ (Hughes and Ellington, 2010) and then generated a library by randomizing the first five base pairs (1:72 to 5:68) in the acceptor stem. Through three rounds of selection, they were able to obtain highly efficient orthogonal tRNA.

#### 3.1.2 Anticodon loop engineering

In certain species, AARS lacks an anticodon binding domain, which is a notable feature observed in several archaeal AARS. Due to the absence of the C-terminal anticodon-binding region, Mj TyrRS is shorter than its counterparts in eukaryotes and bacteria (Kleeman et al., 1997; Steer and Schimmel, 1999). This absence of an anticodon binding domain allows for specific targeting of the anticodon loop, facilitating the reduction of interactions with host AARS while maintaining interactions with the intended AARS partner, ultimately enhancing orthogonality. Particularly, studies indicate that efficiency improvements can be achieved by evolving the anticodon loop for four-base codons. To implement quadruplet codons, a selection involving a four-base codon reporter and a tRNA anticodon loop (32–38 base) library was carried out in *E. coli* (Magliery et al., 2001). This approach identified several efficient four-base codons and demonstrated the potential for extension to five or even six bases. More recently, the Söll group discovered efficient four-base decoding tRNA (qtRNA) through phage-based library generation and selection (Debenedictis et al., 2022). They created an NNNN library for the anticodon of 20 *E. coli* tRNAs and, through phage screening, identified efficient qtRNAs for each amino acid. Furthermore, they developed a library for the anticodon loop and conducted phage-based tRNA evolution to enhance translation efficiency.

#### 3.1.3 Acceptor stem and anticodon loop engineering

In the pursuit of tRNA molecules exhibiting elevated orthogonality and translation efficiency, researchers frequently employed a combined sequential or simultaneous screening approach for the acceptor stem and anticodon loop rather than conducting separate screenings. In order to diversify suppression codons in *E. coli*, researchers used the *Methanobacterium thermoautotrophicum* LeuRS/*Halobacterium sp*. tRNA^Leu^ pair, encompassing opal and quadruplet codons (Anderson and Schultz, 2003). By screening the acceptor stem library, they identified the most active and selective suppressor tRNA, and by using the anticodon loop library, they converted amber to a four-base codon. While the efficiency of opal and AGGA may be lower when compared to amber, the study demonstrated the potential for a broad range of AARS/tRNA and suppressor combinations. In a subsequent study, the same group successfully incorporated ʟ-homoglutamine into proteins using the *Pyrrococcus horikoshii* (Ph) LysRS/tRNA^Lys^ pair with a quadruplet (Anderson et al., 2004). They carried out the evolution of the acceptor stem and anticodon loop to improve the efficiency and orthogonality of qtRNA by employing a similar strategy as in previous research. They demonstrated the incorporation of various proline analogs into *E. coli* using the Ph ProRS/*Archaeoglobus fulgidus* tRNA^Pro^ pair (Chatterjee et al., 2012). They developed orthogonal tRNAs bearing amber (CUA), AGGG, and CUAG anticodons, achieving high suppression yields, particularly with the CUA anticodon.

#### 3.1.4 Anticodon stem/loop engineering

Mutations in the anticodon stem/loop prove effective for enhancing incorporation efficiency, particularly in the context of quadruplet codon suppression. For instance, *N*-tert-butoxycarbonyl-ʟ-lysine was successfully incorporated into green fluorescent proteins in animal cells using the *Methanosarcina mazei* (Mm) PylRS/tRNA^Pyl^ pair through quadruplet codon suppression (Niu et al., 2013). They conducted screening of the anticodon loop library followed by the anticodon stem library, resulting in the identification of a highly efficient orthogonal tRNA. In another study, dual-labeling with UAAs was attempted in *E. coli* using both amber and quadruplet suppression (Wang et al., 2014). They had previously evolved an orthogonal ribosome called riboQ for quadruplet codon decoding (Neumann et al., 2010). In this study, they attempted to enhance the efficiency of riboQ through tRNA evolution. Through screening the anticodon stem/loop library, an orthogonal tRNA^Pyl^_UCUA_ was identified. They successfully achieved dual-labeling with BODIPY-FL and BODIPY-TMR-X on calmodulin and observed fluorescence resonance energy transfer (FRET) in the presence of the calcium using the engineered PylRS/tRNA^Pyl^ pair. The generation of decoding tRNA for UAGN was also achieved through anticodon stem/loop engineering (Wang et al., 2016). The anticodon stem/loop library revealed several orthogonal tRNAs. Notably, UAGU, displaying the highest efficiency, was theorized to induce tRNA distortion due to the presence of “U,” resulting in a +1 frameshift at the ribosome’s P site.

#### 3.1.5 Acceptor stem and T stem engineering

Guided by the structure of the Cys-tRNA^Cys^/EF-Tu complex (Nissen et al., 1999), researchers conducted screening of the acceptor stem and T stem libraries, focusing on regions known to interact with EF-Tu (Guo et al., 2009). Through positive/negative selection on the T stem region (49–53, 61–65 nucleotides) and subsequently on the acceptor stem region (2, 3, 6, 7, 66, 67, 70–71 nucleotides), they successfully improved the UAA incorporation efficiency using the Mj TyrRS/tRNA^Tyr^ pair. Through the evolution of tRNA^Sec^, efficient incorporation of selenocysteine was achieved in bacteria (Thyer et al., 2015). To improve tRNA interaction with both EF-Tu and selA, a library was created by targeting the final base pair of the tRNA acceptor stem and the first two base pairs of the T stem. The newly evolved tRNA^Sec^ exhibited significantly enhanced activity in the canonical translation of selenocysteine.

#### 3.1.6 Variable loop engineering

Utilizing structural distinctions among archaeal AARSs, mutually orthogonal PylRS/tRNA^Pyl^ pairs were identified (Willis and Chin, 2018). The N-terminal domain of Mm PylRS and *Methanosarcina barkeri* (Mb) PylRS, which is absent in *Methanomethylophilus alvus* (Ma) PylRS, is known to bind to the T arm and the variable loop of tRNA (Suzuki et al., 2017). Based on this information, they hypothesized that evolving the variable loop of Ma tRNA^Pyl^ could produce an orthogonal tRNA^Pyl^ for Mb PylRS/tRNA^Pyl^. To achieve this, they first created a library for the three positions of the variable loop, which led to the successful generation of an orthogonal Ma tRNA^Pyl^ for Mb PylRS/tRNA^Pyl^. They then extended the variable loop’s length to six nucleotides and conducted screening, resulting in the discovery of novel orthogonal PylRS/tRNA^Pyl^ pairs. Subsequently, employing a similar strategy, the same group successfully identified triply orthogonal PylRS/tRNA^Pyl^ pairs by screening a variable loop library of the tRNA (Dunkelmann et al., 2020).

#### 3.1.7 Anticodon stem and acceptor stem/loop engineering

The Sc TrpRS/tRNA^Trp^ pair was evolved to increase its efficiency using a newly developed evolution method called compartmentalized partnered replication (CPR) (Ellefson et al., 2014). In this process, the anticodon stem and acceptor stem/loop tRNA libraries were created and then co-evolved with AARS to improve the efficiency of 5-hydroxy-ʟ-tryptophan incorporation. After conducting 10 rounds of CPR, seven effective AARS/tRNA pairs were identified. Among these pairs, the tRNA with loop mutations U16G, G43U, and U58G demonstrated the highest efficiency.

#### 3.1.8 Acceptor stem, T stem/loop, and anticodon stem/loop engineering

Mj tRNA variants were developed using positive/negative selection based on CPR and a mutant phenylalanyl-tRNA synthetase, leading to the discovery of hits with high suppression efficiency and significantly reduced background (Maranhao and Ellington, 2017). In this study, mutations were introduced into various regions, encompassing the acceptor stem, the T stem/loop, and the anticodon stem/loop, to facilitate the incorporation of 3-halo-tyrosines into proteins in *E. coli*.

#### 3.1.9 Virus-assisted directed evolution strategy

Recently, researchers have employed a virus-mediated gene delivery technique to conduct directed evolution of tRNA within animal cells called virus-assisted directed evolution strategy (VADER) (Jewel et al., 2023; Jewel et al., 2024). They employed a selection system diverging from the commonly used positive/negative selection method. Their library screening method consists of two steps. The first selective amplification step involves removing inactive tRNA by using plasmid encoding amber mutant of the genes necessary for AAV replication. In the second step, they used an azide handle for labeling and pull-down to retain only the active and orthogonal tRNA. This innovative approach has led to a notable enhancement in the efficiency of unnatural amino acid incorporation in mammalian cells by overcoming the limit of the library size.

### 3.2 Rational approaches to tRNA engineering

A directed evolution approach based on a random library can often be time-consuming and labor-intensive since its effectiveness is directly proportional to the number of entries. In order to ensure the profitability of the evolution process, it is crucial to manage a larger-scale library. Researchers have therefore attempted to employ a rational design of tRNA based on diverse information including the tRNA structure, tRNA-binder complex structure, sequences of tRNA/AARS pairs from various origins, and the identity elements of each tRNA. This approach has helped to reduce the associated time and costs and expedite the evolution process.

#### 3.2.1 Designing tRNA based on structure information

**FIGURE 7:**
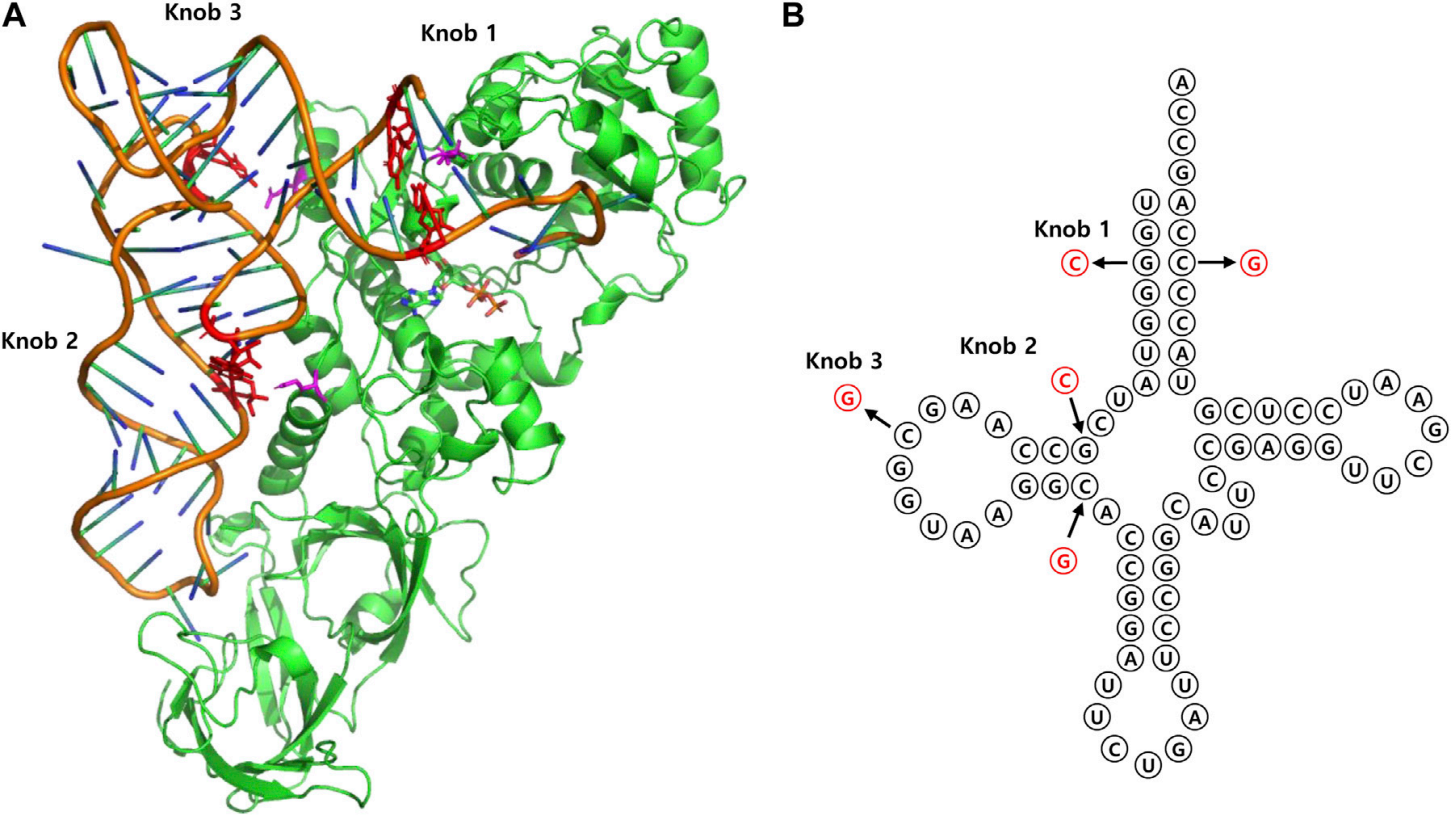
**(A)** 3D structure of *E. coli* GlnRS—tRNA^Gln^ complex (PDB entry 1GTR). Three interaction sites on tRNA are represented as “knobs.” **(B)** Cloverleaf representation of each knob site.

The fundamental approach to rational design involves analyzing the sequence and structure of tRNA, as well as the structure of tRNA binding sites in proteins that interact with it. A notable example is the evolution of Ec GlnRS and tRNA^Gln^, whose complex structure was resolved at high resolution through X-ray crystallography (Rould et al., 1991). Based on the structure, researchers developed orthogonal amber suppressor tRNA by rationally mutating selected tRNA basses. To prevent recognition by the existing GlnRS, they applied various mutations to the three knobs of tRNA believed to interact with GlnRS in the elucidated structure. Concurrently, the anticodon was modified to amber, resulting in the generation of an orthogonal suppressor tRNA (Liu et al., 1997a; Liu et al., 1997b) (Figure 7). However, for the precise design of mutations, a thorough understanding of the intricate complex structures of tRNA and its binding partner is essential, posing a significant challenge. Therefore, researchers have devised various strategies to implement rational design efficiently, outlined as follows.

#### 3.2.2 Adopting tRNA across different origins

Distinct identity elements exist in different tRNAs from various species. This implies that natural AARS enzymes from one species may not recognize tRNAs derived from another species. This cross-species orthogonality of tRNA and AARS has been harnessed to discover and utilize orthogonal AARS/tRNA pairs. For example, it was discovered that not only Sc tRNA^Gln^ but also its amber suppressor mutant tRNA^Gln^_CUA_ was not charged by Ec GlnRS (Whelihan and Schimmel, 1997). Furthermore, it was demonstrated that Sc GlnRS can aminoacylate Sc tRNA^Gln^_CUA_ mutants within *E. coli* while still maintaining orthogonality to the endogenous *E. coli* tRNA^Gln^. Expanding on these discoveries, the introduction of UAA into the target protein in *E. coli* was ultimately achieved using the Sc GlnRS mutant (Liu and Schultz, 1999). Similarly, an orthogonal pair, Mj TyrRS/Mj tRNA^Tyr^_CUA_, derived from *M. jannaschii*, was developed to function orthogonally within *E. coli* (Wang et al., 2000). The orthogonal Mj tRNA^Tyr^_CUA_ features a C1:G72 base pair, considered a crucial negative identity element that Ec TyrRS cannot recognize. This C1:G72 base pair is also present in eukaryotic tRNA^Tyr^, such as those found in yeast or humans. Exploiting these characteristics, Ec TyrRS and Ec tRNA^Tyr^_CUA_ were employed as an orthogonal pair for amber suppression in yeast and mammalian cells (Edwards and Schimmel, 1990; Chin et al., 2003). In another study, an orthogonal pairing was established using Ec LeuRS and Ec tRNA^Leu^_CUA_, facilitating the effective incorporation of UAAs in yeast (Wu et al., 2004).

#### 3.2.3 Rationally engineering tRNA for improving efficiency and orthogonality

Going beyond the mere adoption of tRNAs from diverse species, researchers have sought to introduce mutations into tRNAs to enhance their expression levels, stability, orthogonality, suppression capability, and interaction with binding partners such as AARS or EF-Tu. This process is guided by existing knowledge of tRNA sequences and informed by structural and biochemical information. For example, efforts have been made to carry out amber suppression using the Sc TrpRS/Sc tRNA^Trp^_CUA_ pair derived from *S. cerevisiae* in *E. coli* (Hughes and Ellington, 2010). However, undesired amber suppression was observed in *E. coli* when expressing only Sc tRNA^Trp^_CUA_ without Sc TrpRS. Sequence analysis of the expressed protein revealed that the endogenous LysRS in *E. coli* was acylating lysine onto Sc tRNA^Trp^_CUA_, generating this unintended outcome. In order to establish orthogonality with Ec LysRS, a new Sc tRNA^Trp^_CUA_ was designed by identifying crucial elements of Ec tRNA^Lys^ and removing them from Sc tRNA^Trp^_CUA_. The introduction of multiple GC pairs into the acceptor stem to enhance stem stiffness and the incorporation of U30-G40 wobble to decrease tRNA-AARS interaction significantly impeded Ec LysRS recognition. Subsequently, in an attempt to enhance amber suppression and UAA incorporation efficiency in the Sc TrpRS/Sc tRNA^Trp^_CUA_ system, a further study involved introducing a library into the acceptor stem of Sc tRNA^Trp^_CUA_, which proved successful (Chatterjee et al., 2013b).

In an effort to enhance the yield of UAA incorporation using the Mj PylRS/tRNA^Pyl^ pair, researchers focused on optimizing the interaction between tRNA^Pyl^ and *E. coli* EF-Tu (Fan et al., 2015). They hypothesized that the T stem of tRNA^Pyl^ would play a pivotal role in EF-Tu binding, based on the established binding structure of *E. coli* tRNA and *Thermus aquaticus* EF-Tu (Nissen et al., 1999). Using this insight, they systematically designed mutations and conducted targeted screening at specific positions in tRNA^Pyl^. This approach resulted in the generation of an optimized version, tRNA^Pyl^ opt, which exhibited improved efficiency in UAA incorporation.

The Söll group made a notable discovery revealing that in certain archaea, the charging process of tRNA^Cys^ with cysteine follows a distinctive pathway, divergent from the conventional CysRS-based direct charging pathway (Sauerwald et al., 2005). Instead, a specific aminoacyl-tRNA synthetase known as phosphoseryl-tRNA synthetase (SepRS) takes the initial step by charging tRNAcys with phosphoserine (Sep). Subsequently, the resulting Sep-tRNA^Cys^ undergoes conversion into Cys-tRNA^Cys^ facilitated by Sep-tRNA:Cys-tRNA synthase (SepCysS) (Yuan et al., 2006). Taking advantage of this unique pathway, the group engineered tRNA^Cys^ to incorporate Sep into proteins in *E. coli*, utilizing *Methanococcus maripaludis* (Mmp) SepRS and Mj tRNA^Cys^ (Park et al., 2011). The rational design of orthogonal tRNA was implemented utilizing insights from their prior study (Hohn et al., 2006), which had identified the essential tRNA identity elements for Sep charging through both evolutionary and experimental perspectives. In particular, the introduction of a C20U mutation into the existing Mj tRNA^Cys^ played a pivotal role in enhancing the Sep charging efficiency of the amber version Mj tRNA_CUA_ by SepRS.

#### 3.2.4 Tailoring tRNA for UAA incorporation in mammalian cells

**FIGURE 8:**
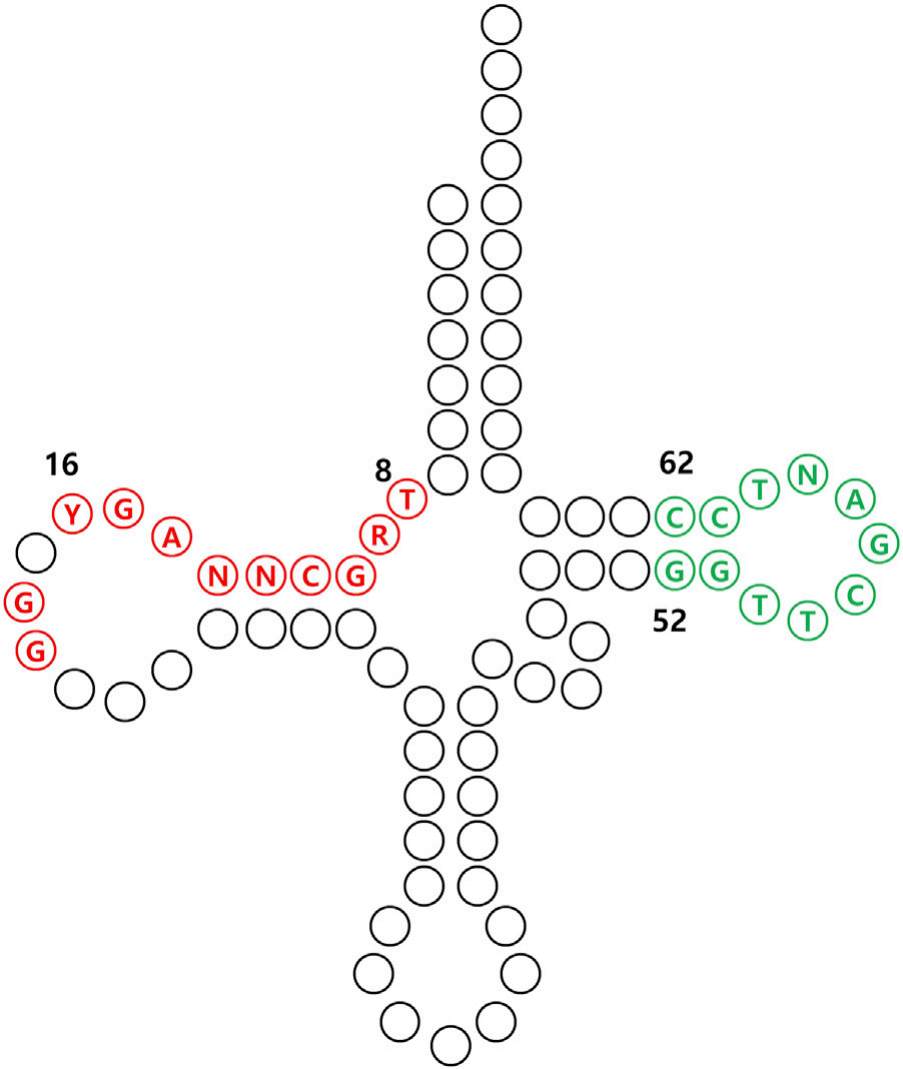
Conserved internal promoter sequences of tRNA in mammalian cells. The A-box, represented by the sequence TRGCNNAGY for positions 8 to 16, along with G18 and G19, and the B-box, indicated by GGTTCGANTCC for positions 52 to 62 are marked in red and green, respectively.

To achieve efficient expression of tRNAs in mammalian cells, it is essential to incorporate an internal promoter sequence within the tRNA (Figure 8). This internal promoter sequence then facilitates subsequent processes, including amber suppression and eventual incorporation of UAAs. For example, efficient incorporation of UAAs was observed in CHO cells when *Bacillus stearothermophilus* tRNA^Tyr^_CUA_, containing an internal promoter sequence, was utilized (Sakamoto et al., 2002). In another instance, mutations were introduced into *B*. *subtilis* tRNA^Trp^_UCA_, using the pseudo-A-box sequence and the 5′ flanking sequence from the *Arabidopsis thaliana* Trp-1 gene, with the aim of enhancing the efficiency of UAA incorporation (Zhang et al., 2004).

In a similar context, one study aimed to utilize Mm tRNA^Pyl^ as a framework for gaining insights into the optimal tRNA sequence for suppression in mammalian cells (Serfling et al., 2018). They first explored various conserved sequences present in human tRNA sequences, and then introduced each conserved sequence into Mm tRNA^Pyl^ and checked the suppression efficiency in mammalian cells. Consistent with prior research, the significance of the internal promoter sequence in tRNA for effective amber suppression in mammalian cells was underscored, presumably linked to improved tRNA expression levels. Moreover, the positive impact of G:C pair mutations in the stem region on suppression was identified, contributing to enhanced tRNA stability in the cytosol. Additionally, similar to strategies employed in bacterial systems, attempts were made to introduce mutations into the tRNA’s EF binding region to further enhance efficiency (Fan et al., 2015).

#### 3.2.5 Customizing tRNA for UAA incorporation at multiple sites

Achieving multiple-site incorporation of UAA relies crucially on enhancing suppression efficiency and establishing new orthogonality. To establish an optimized system for such incorporation across multiple sites, an attempt was made to mutate the G:U wobble pair in the tRNA stem region, substituting it with a canonical G:C base pair (Chatterjee et al., 2013a). This concept was inspired by the hypothesis that reducing wobble pairs in the tRNA stem enhances suppression efficiency by increasing tRNA stability during protein expression processes (Anderson and Schultz, 2003). Introducing the canonical base pair resulted in an enhanced yield of UAA incorporation at multiple sites in *E. coli*. In another study aimed at achieving multiple-site UAA incorporation in mammalian cells, researchers sought to create a new orthogonal AARS/tRNA pair by utilizing PylRS/tRNA^Pyl^ derived from the gut microbiome *Candidatus Methanomethylophilus alvus* Mx1201, which is originally orthogonal to the Mm PylRS/tRNA^Pyl^ system (Meineke et al., 2018). Similar to the prior research, the initial step involved the removal of the wobble pair. Additionally, the study emphasized the pivotal role of Mm PylRS’s N-terminus in recognizing Mx1201 tRNA^Pyl^. The variable loop of Mx1201 tRNA^Pyl^ was rationally designed based on the known structures of Mm PylRS N-term domain and tRNA^Pyl^. This rational design approach led to the identification of a new PylRS/tRNA^Pyl^ pair capable of successfully achieving orthogonal multiple-site incorporation.

In an attempt to find two distinct mutually orthogonal AARS/tRNA pairs for multiple site incorporation, using the initiator tRNA was suggested. In *E. coli*, protein expression is initiated by Ec tRNA^fMet^. By introducing the Mj tRNA^Tyr^ identity element to Ec tRNA^fMet^ and changing its anticodon from CAU to CUA, various UAAs can be incorporated at the first position of the protein when Mj TyrRS is co-expressed (Tharp et al., 2020). Furthermore, it was found that simultaneous expression of orthogonally designed Ec tRNA^fMet^_CUA_ (*i*tRNA^Ty2^_CUA_)/Mj TyrRS pair with Ma PylRS/tRNA^Pyl^_UUA_ pair can successfully incorporate two different UAAs into the first and second amino acid positions of a single protein. In the subsequent study, they successfully incorporated three distinct UAAs in a single polypeptide using three different pairs of Mj TyrRS/*i*tRNA^Ty2^_AUA_, Ma PylRS/tRNA^Pyl^_CUA_, and Mm PylRS/tRNA^Pyl^_UUA_ that were orthogonal to each other (Tharp et al., 2021).

#### 3.2.6 Crafting a chimeric tRNA

The development of chimeric tRNAs involves a rational design strategy that integrates diverse motifs from two or more tRNAs originating from distinct sources into a unified tRNA molecule. This allows the tRNA to interact with a range of binding partner proteins, such as AARS or EF-Tu, potentially facilitating the development of novel orthogonality. The incorporation of selenocysteine, the 21st natural amino acid in biological systems, deviates from the conventional AARS-tRNA pathway. SerRS initially charges serine onto tRNA^Sec^ (Leinfelder et al., 1988). Subsequently, the tRNA-charged serine undergoes conversion into selenocysteine by SelA. The resulting selenocysteyl-tRNA^Sec^ actively participates in protein expression processes with the assistance of a specialized elongation factor called SelB. SelB recognizes a specific mRNA structure known as the selenocysteine insertion sequence (SECIS) element, facilitating the incorporation of selenocysteine. In an effort to circumvent SECIS element dependency during selenocysteine incorporation, the Söll group explored the use of the EF-Tu system (Aldag et al., 2013). To achieve this, they developed a chimeric tRNA named tRNA^Sec^ UTu, derived from the scaffold of tRNA^Ser^ and incorporating the acceptor stem sequence of tRNA^Sec^. While maintaining recognition by SelA, this chimeric structure successfully incorporates selenocysteine through EF-Tu.

**FIGURE 9:**
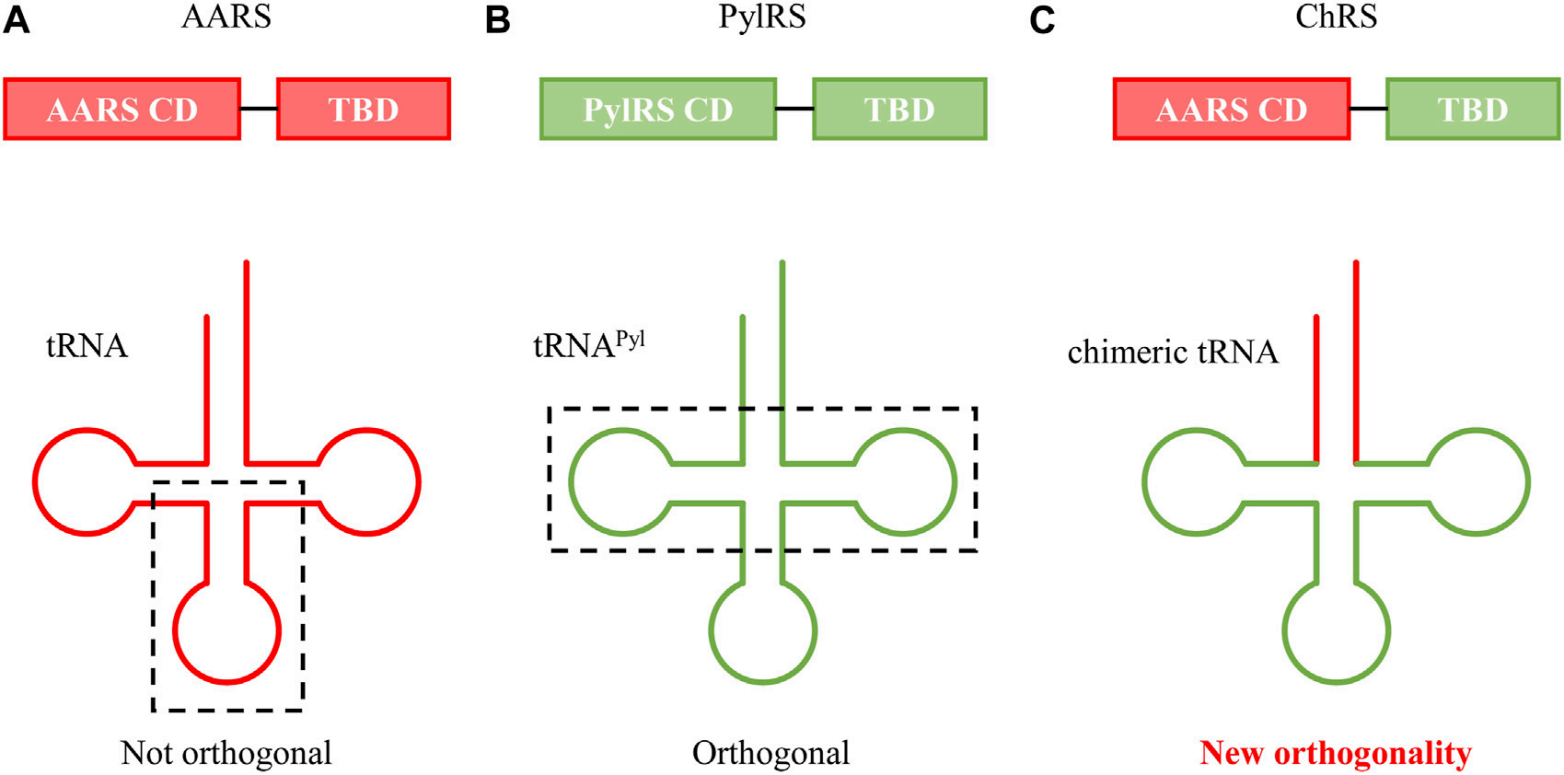
Cartoon depictions of **(A)** AARS/tRNA, **(B)** PylRS/tRNA^Pyl^, and **(C)** chimeric AARS/tRNA (ChRS/ChT) pairs. CD and TBD denote the catalytic domain and tRNA binding domain, respectively.

In mammalian cells, the synthesis of proteins in mitochondria involves a distinct set of tRNAs that are orthogonal to those present in the host cell. Inspired by this phenomenon, researchers incorporated a PylRS recognition sequence into mitochondrial tRNA, creating a chimeric orthogonal tRNA in mammalian cells (Serfling et al., 2018). This chimeric approach has also been employed to create novel orthogonal AARS/tRNA pairs suitable for both eukaryotic and prokaryotic systems. For instance, in a study where a chimeric AARS mutant was developed by combining the tRNA binding domain of PylRS with the catalytic domain of Ec HisRS, researchers utilized a chimeric tRNA based on tRNA^Pyl^ (Ding et al., 2020). (Figure 9) This chimeric tRNA, featuring only a partial replacement of the acceptor stem with Ec tRNA^His^, facilitated recognition and UAA charging by the chimeric AARS. The versatility of this chimeric system was demonstrated in both *E. coli* and mammalian cells for efficient UAA incorporation. Advancing further, an effort was made to introduce a library into the acceptor stem of these chimeric tRNAs, aiming to improve the overall efficiency of incorporation (Zhao et al., 2021).

#### 3.2.7 The selenocysteine adventure

The development of the previously mentioned tRNA^Sec^ UTu represents a significant achievement as it allows SECIS-independent selenocysteine incorporation at the amber codon (Aldag et al., 2013). However, a drawback of this system is its inadequate recognition by SelA, leading to substantial serine misincorporation due to insufficient conversion of serine to selenocysteine. To overcome this limitation, the Söll group sought to improve tRNA^Sec^ UTu by designing a new tRNA, tRNA^Sec^ UTuX (Miller et al., 2015) based on the co-crystal structure of *Aquifex aeolicus* SelA and *Thermus tengcongenisis* tRNA^Sec^ (Itoh et al., 2013). tRNA^Sec^ UTuX not only enhanced SelA-mediated conversion compared to the original tRNA^Sec^ UTu but also improved serine charging efficiency by SerRS, resulting in an increase in the yield of selenoprotein synthesis. Addressing the same issue, the Ellington group adopted a different strategy. tRNA^Sec^ Ux was redesigned by introducing EF-Tu recognition elements into tRNA^Sec^, leading to enhanced selenocysteine incorporation through EF-Tu (Thyer et al., 2015). Another group attempted to modify the acceptor stem, T stem, and base 59 on the T loop of tRNA^Sec^ UTu to enhance the EF-Tu binding affinity of selenocysteine-charged tRNA in comparison to serine (Fan et al., 2018). Individual and combined mutations were introduced at each site, leading to the identification of tRNA^Sec^ UTu with a singular mutation at base 59, demonstrating the highest selenocysteine incorporation. The enhancement in incorporation is attributed to the reinforcement of the interaction between the T loop and the D loop, crucial for maintaining the L-shaped three-dimensional structure of tRNA. This tRNA variant, tRNA^Sec^ UTuT6, exhibited significantly superior selenocysteine-specific incorporation compared to tRNA^Sec^ UTu, reaching levels comparable to tRNA^Sec^ UTuX and tRNA^Sec^ Ux.

**FIGURE 10:**
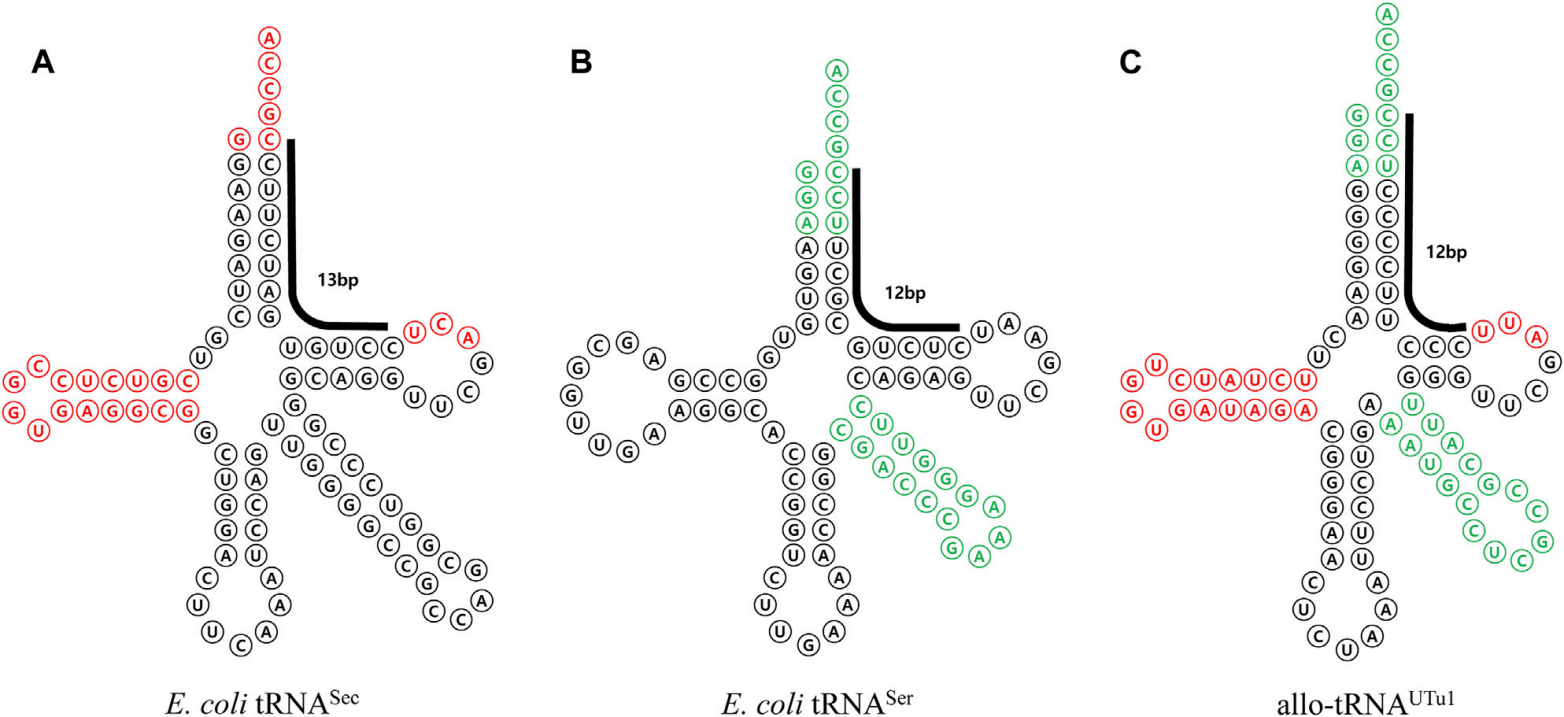
Allo-tRNA for selenocysteine incorporation mediated by EF-Tu. **(A)** *E. coli* tRNA^Sec^ sequence and structure. *E. coli* SelA recognition sequences are shown in red. The 13-base pair SelB recognition sequences, spanning the acceptor stem and T stem are denoted by a bold line. **(B)** *E. coli* tRNA^Ser^ sequence and structure. *E. coli* SerRS recognition sequences are shown in green. The 12-base pair that is more readily recognized by *E. coli* EF-Tu is indicated by a bold line. **(C)** Structure and sequence of allo-tRNA, designed and engineered by combining elements from *E. coli* tRNA^Sec^ and *E. coli* tRNA^Ser^, for the multiple incorporation of senenocysteine.

The tRNA^Sec^ variants mentioned so far depend on *E. coli* SelA to change Ser to Sec. Ec SelA recognizes a 13 bp sequence that extends from the acceptor stem to T stem, which is an essential element for SelA recognition. This means that all the EF-Tu-mediated variants carry a 13 bp branch structure. Thus, these tRNAs can also be recognized by the SECIS/SelB system, making it difficult to achieve completely independent expression. To address this, the Söll group developed a new system for Sec incorporation using *Aeromonas salmonicida* SelA, which recognizes only tRNA possessing a total of 12 base pairs in the acceptor stem and T stem and allo-tRNA variants that were discovered in metagenomic origin (Mukai et al., 2018). Various allo-tRNA variants were created by adopting the D loop portion from As tRNA^Sec^. By creating various allo-tRNA variants, incorporating the D loop portion from As tRNA^Sec^, and optimizing SelA and allo-tRNA^Sec^ expression levels using various promoters, a successful system for efficient multiple-site Sec incorporation was established (Figure 10). Further study demonstrated the feasibility of selenoprotein production in yeast through the optimization of As SelA using modified yeast tRNA (Hoffman et al., 2023). For a more in-depth exploration of selenoprotein biosynthesis, a comprehensive review is available (Wright and O’Donoghue, 2023).

## 4 Conclusion

Efficient orthogonal AARS/tRNA pairs identified in *E. coli* often exhibit diminished efficacy when applied to higher organisms. To overcome this challenge, the directed evolution of tRNA alongside AARS engineering has emerged as a strategy to enhance GCE efficiency. The typical approach to tRNA evolution has been implemented by screening a tRNA library. This method provides the opportunity to obtain highly efficient tRNA by selecting an appropriate randomizing region. However, the library screening method faces limitations, particularly in the context of animal cells or multicellular organisms, due to its restricted library size. This emphasizes the need for tRNA engineering using rational approaches. In theory, employing rational design allows for the acquisition of tRNA with high orthogonality and stability within cells, regardless of host cell type. Further structural information and biochemical research data on tRNA will accelerate the advancement of rational design methods for tRNA. Furthermore, the utilization of machine-learning algorithms to achieve precise prediction of protein-tRNA structure will advance the tRNA rational design strategies, facilitating the exploration of new orthogonal AARS-tRNA pairs. The integration of rational design and library screening approaches offers a comprehensive strategy for achieving success in tRNA evolution, contributing to the further diversification and ease of use of GCE in the future. As researchers refine tRNA engineering techniques, the horizons of GCE will undoubtedly broaden, revealing new opportunities for scientific exploration and technological breakthroughs.
